# A Spatio-Temporal Attention Mechanism Based Approach for Remaining Useful Life Prediction of Turbofan Engine

**DOI:** 10.1155/2022/9707940

**Published:** 2022-10-14

**Authors:** Cheng Peng, Jiaqi Wu, Zhaohui Tang, Xinpan Yuan, Changyun Li

**Affiliations:** ^1^School of Computer Science, Hunan University of Technology, Zhuzhou 412000, China; ^2^School of Automation, Central South University, Changsha 418003, China

## Abstract

The time-series data generated by turbofan engines has a great degree of complexity and dynamics. At present, recurrent neural networks are commonly used to model and forecast the remaining useful life (RUL). The relationship of the sample data is not taken into account, and there are issues such as gradient explosion. In view of this, a spatio-temporal attention model is proposed, which comprehensively relates to the temporal association of data features and the hidden state of data features in space. At the same time, position coding is performed on the temporal relationship, avoiding the use of recurrent neural networks. Experimental results show that by combining the two dimensions, the predictive performance of the model is significantly improved. Compared with different methods on the four data sets of the commercial modular aerospace propulsion system simulation (C-MAPSS), the stability and prediction accuracy of the spatio-temporal attention model are better than that of alternative methods.

## 1. Introduction

Among the functions of failure prediction and health management, RUL prediction is the core function. The turbofan engine is one of the key power equipment of the aircraft, and its safe and stable work is extremely crucial. When the accuracy of the turbofan engine RUL prediction reaches a certain level, the original regular maintenance can be turned into predictive maintenance, which will increase the overall use time of the equipment, avoid irreparable deterioration of the equipment, and reduce the expenditure of human and material resources. Therefore, it is extremely essential to improve the RUL prediction accuracy of turbofan engines.

At present, as a data-driven method, deep learning is widely used for RUL prediction. Regarding the research on RUL prediction of turbofan engines, deep learning methods can be divided into methods based on recurrent neural networks [1], convolutional neural networks [2], and hybrid model methods.

The methods based on the recurrent neural network are to use the recurrent neural network to process the time relationship of the sequence data and predict the RUL of the turbofan engine. In order to improve the accuracy of RUL prediction, Heimes [3] proposed a prediction method based on recurrent neural network (RNN), which uses time gradient calculation and extends Kalman filter for training, and predicts the RUL of turbofan engines. However, RNN has the problem of gradient disappearance. In order to predict RUL stably in an environment of complex operation, mixed faults, and strong noise, Yuan et al. [4] established a long short-term memory (LSTM) neural network model for fault diagnosis and RUL prediction of turbofan engines. But LSTM can only infer the feature relationship from one direction of the time series. In order to discover the long-term dependence relationship of sensor time series signals, Wu et al. [5] designed an RUL prediction method based on deep long short-term memory (DLSTM) network using multiple sensor time series signals. However, DLSTM has the problem of long calculation time. In order to solve the problem, the generated vector contribution of each time step pair of RNN on the autoencoder is equal, Duan et al. [6] adopted a method of assigning weight to each time step through an attention mechanism to improve the prediction ability of BiGRU. However, the model does not consider the relationship between the sensor's positions in space. Aiming at the problem of complex features of sensor data in RUL prediction, Muneer et al. [7] applied an LSTM model based on the attention mechanism to achieve accurate RUL prediction. But recurrent neural networks cannot achieve parallel computing. A compromise in performance against computational efficiency is found to be unfavorable to eeting practical needs [8].

The methods based on the convolutional neural network are to use the convolutional neural network to extract the local features of the sequence data and then predict the RUL. Wen et al. [9] established a new residual convolutional neural network (ResCNN) to solve the problem of gradient disappearance and predict the RUL of turbofan engines but did not consider the relationship between the sensors at each time step. In order to solve the problem of model adaptation to different data sources, Li et al. [10] proposed a domain adaptive RUL prediction by integrating adaptive batch normalization (AdaBN) into a deep convolutional neural network (DCNN) model, but mapping the features of different data sources to the same feature relationship space is still a problem. In view of the limited ability of traditional data-driven methods in extracting complex features, Li et al. [11] designed a multiscale convolutional neural network (MSCNN) to directly establish the relationship between monitoring data and RUL, but the size of the multiscale convolution kernel is determined empirically.

The hybrid model method combines the advantages of several methods. In order to make full use of the advantages of CNN and LSTM models, Borst [12] proposed a method combining LSTM and CNN to predict the RUL of turbine engines. Remadna et al. [13] use CNN with bi-directional long short-term memory (BDLSTM) networks where CNN extract spatial features while BDLSTM extracts temporal features. Chen et al. [14] combines the DCNN and the BDLSTM was used to train the source domain and target domain data simultaneously to extract the common features of the equipment under the condition of sufficient samples. Jiang et al. [15] propose a data-driven method based on BDLSTM and multiscale convolutional neural network (MSCNN) for RUL estimation. But how to effectively splice CNN and LSTM models is currently a problem that needs to be resolved.

In conclusion, a lot of research has made great progress on the RUL prediction of turbofan engines. However, the neural network still has the following problems in RUL prediction. Firstly, the recurrent neural network can only infer the internal information of the time series from one direction or two directions, but does not consider the relationship between each time tick, and cannot achieve parallel operations. Secondly, for observational data, most studies only consider how to predict RUL in terms of time while ignoring the spatial connection of data features that also assists in the prediction of RUL. Last but not least, for long-term sequences, the recurrent neural network has the problem of gradient disappearance or gradient explosion. Therefore, how to avoid the gradient problem while also taking into account the position of the time series is also a problem.

In view of the aforementioned shortcomings, this paper proposes a spatio-temporal attention model to predict RUL. The main contributions are as follows. First, from the perspective of time relationships, a time position coding method is designed to code the position of the time series and actively apply the time series relationship to the model. Secondly, in order to overcome the problem of incomplete information on the time dimension and the inability of parallel computing of recurrent neural network, an attention mechanism is used to extract the state relationship of features in the two dimensions of time and space, respectively. Finally, a convolution module is designed, which combines the time feature map and the space feature map, and uses two fully connected layers to predict the RUL of the turbofan engine. All in all, the attention mechanism solves the problem that recurrent neural networks cannot perform parallel computation, and the model does not take into account the spatial relationship of features or the different weights of each timestamp. In order to verify the effectiveness of the method, we made predictions on 4 datasets of NASA's C-MAPSS.

The rest of the paper is organized as follows. The second part introduces the methodology of spatio-temporal attention, including data preprocessing, and spatio-temporal attention models; the third part analyzes the experimental results.

## 2. Materials and Methods

### 2.1. The Procedure of Remaining Useful Life Prediction

**Figure 1:**
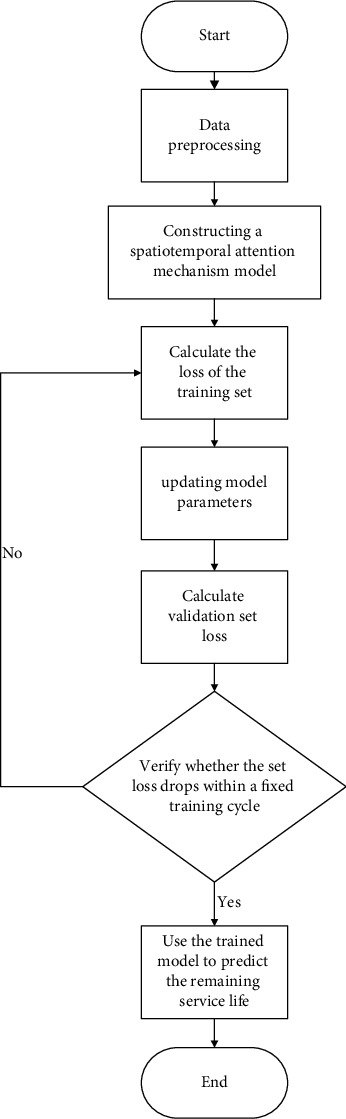
RUL forecast flow chart.

It is shown in Figure 1 that the procedure of RUL prediction includes the following 4 steps.

1. Data preprocessing: the data in the original data set contains redundant features, and the unit scales between the data are inconsistent, so it is necessary to preprocess the data and then standardize it.
2. Data set division: the whole training set is divided into a training set and verification set by early stop strategy. The training set is used to adjust the parameters of the model during model training; the verification set is used to train the early stop model. That is, in 8 training cycles, if the overall RMSE of the verification set does not decline, the training will be terminated.
3. Model Construction: build a spatio-temporal attention mechanism model, and in the processing time dimension, first encode the input data, and then use it as the input of the model.
4. RUL prediction: use the trained model to predict the remaining life on the test set and evaluate the corresponding indicators.

**Figure 2:**
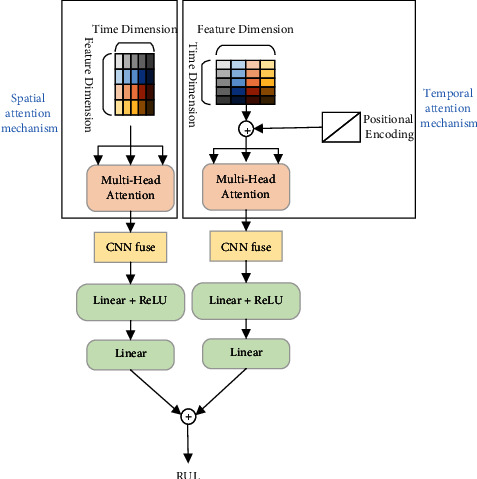
Spatial-temporal attention mechanism model structure.

The structure of the spatial-temporal attention mechanism model is shown in Figure 2. As can be seen from the diagram, the whole structure can be divided into spatial attention mechanism, temporal attention mechanism, convolution layer fusion, and full connection layer. Spatial attention mechanism and temporal attention mechanism modules obtain the spatial and temporal characteristics of the sequence data mainly through the multi-head self-attention mechanism [16]. Convolution layer fusion further extracts features, fuses the feature map into a vector, and reduces the feature dimension. The fully connected layer predicts RUL by eigenvectors. The process and principles of this approach are described in detail below.

### 2.2. Data Preprocessing

**Figure 3:**
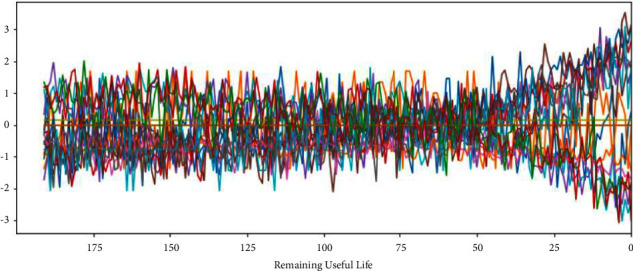
Engine unit 1 sensor data in FD001 sub-dataset.

**Figure 4:**
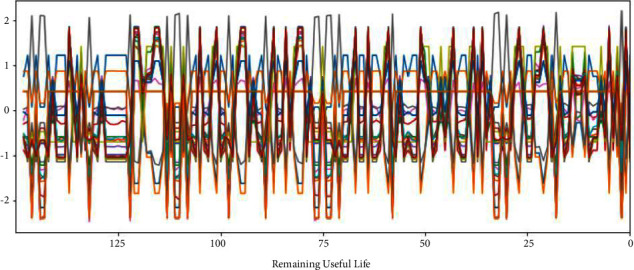
Engine unit 1 sensor data in FD002 sub-dataset.

**Figure 5:**
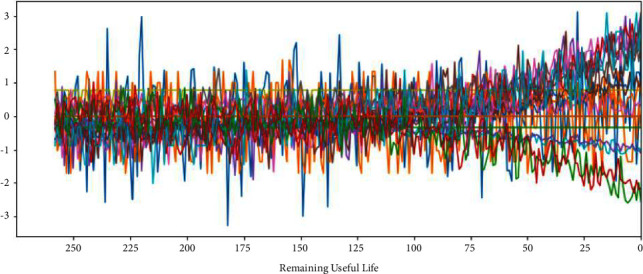
Engine unit 1 sensor data in FD003 sub-dataset.

**Figure 6:**
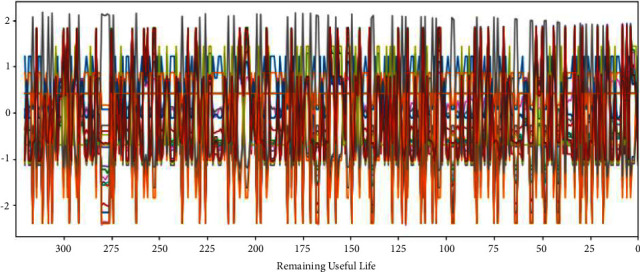
Engine unit 1 sensor data in FD004 sub-dataset.

The C-MAPSS dataset contains three operational settings and 21 sensor data. Figures 3–6 show the standardized 24 index data of Engine Unit 1 of the FD001, FD002, FD003, and FD004 sub-datasets. In these figures, each color represents a feature; RUL stands for the numerical value of abscissa; the ordinate value indicates the magnitude of the characteristic value. The model is able to learn the functional relationship between RUL and characteristic value.

However, some of the data features do not change much over the entire time period, which is not helpful for the prediction of the model. Therefore, it is necessary to select data features that vary over time to predict RUL. A large number of studies have shown that the prognosability method can be used to represent the variability of features. This paper uses the prognosability [17] method to select features. As in the following formula:

(1) $$\begin{array}{c} prognosability=\exp\;\left(-\frac{std_{j}\left(x_{j}\left(N_{j}\right)\right)}{mean_{j}\left|x_{j}\left(1\right){-x}_{j}\left(N_{j}\right)\right|}\right),j=1,\cdots ,M\text{.} \end{array}$$

If a feature's prognosability is equal to 0 or NaN, it is not preserved. Conversely, the autonomy of the model has been improved, and the problem of relying on human experts for feature selection has been addressed [8].

After selecting features, the data needs to be standardized and scaled to the same scale. Z-score [18] can unify data of different magnitudes into the same magnitude, and the conversion process can easily extend to more complex datasets, so the Z-score method is used to standardize the data. As in the following formula:

(2) $$\begin{array}{c} z=\frac{x-u}{\sigma }\text{.} \end{array}$$

*u* is the mean of the selected feature, the standard deviation of the selected feature, and *x* represents the feature value at a given time.

**Figure 7:**
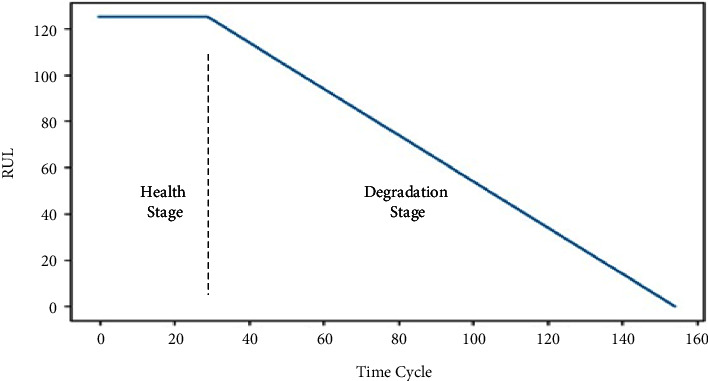
Distribution of turbo engine real life.

The health state of a turbofan engine is generally divided into health state and deterioration state. In a healthy state, the RUL of a turbofan engine is in a constant state. In the degenerate state, its RUL is in a descending state. Therefore, a health threshold needs to be set for turbo engine equipment. The results show that when the initial RUL value in the C-MAPSS dataset is in the range 120–130, the output is stable. Therefore, we set the RUL threshold to 125. See Figure 7 for details.

After these processes, the data also needs to be divided into time windows as input to the model. The input of the model = [*x*_1_, *x*_2_,…, *x*_*f*_], where,

(3) $$\begin{array}{c} x_{i}=\left[\begin{array}{c} x_{i}^{widoow\_start}\\ x_{i}^{widoow\_start+1}\\ \vdots \\ x_{i}^{widoow\_en\;d} \end{array}\right],i=1,\cdots ,f\text{.} \end{array}$$

*f* is the number of time feature selected, (window_end-window_start) is the size of the time window, and the time step is set to 1 for more training samples.

### 2.3. Spatial-Temporal Feature Extraction

**Figure 8:**
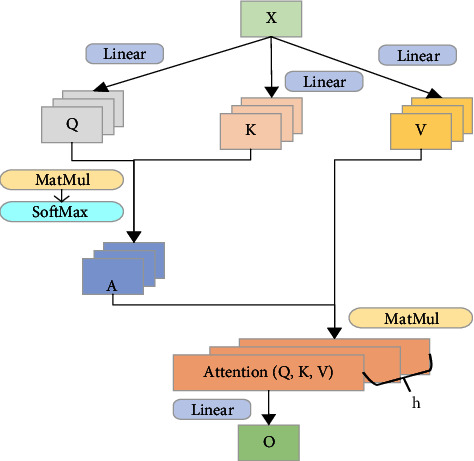
Multi-head attention structure.

Most current studies only consider how to predict RUL in terms of temporal relationships, ignoring the spatial relationships of data. This paper considers the internal relationships between extracting data in both temporal and spatial directions. However, data may have multiple relationships in the same direction. In this paper, multi-head attention [16] is used to extract multiple connections of data in both time and space dimensions while avoiding the problem of gradient disappearance or gradient explosion of circulating nerves and improving the speed of parallel computing. The multi-head attention structure is shown in Figure 8.

Where *X* represents input data; A represents the relationship weight between the row vectors in the input data *x*, which is derived from the matrices *Q* and *K*; *V* represents the specific real value characteristics of the row vector in the input data *X*; *h* represents the number of relationships. The calculation formula of multi-head attention is as follows.

(4) $$\begin{array}{c} Q=XW^{q}\text{,}\\ V=XW_{v}\text{,}\\ K=XW_{k}\text{,}\\ A=\text{softmax}\left(\frac{QK^{T}}{\sqrt{d_{k}}}\right)\text{,}\\ \text{Attention}\left(Q,K,V\right)=AV\text{.} \end{array}$$

When using the attention mechanism method, some current studies usually use the attention mechanism to extract the relationship between the time of the vector, such as the relationship between *t*_1_ time vector and *t*_2_ time vector. Then the time relationship is input into the recurrent neural network to predict RUL, but the recurrent neural network is prone to the problem of gradient disappearance or gradient explosion. If only the attention mechanism is used, the model may ignore the position of the time series. For example, it does not consider that the vector at *t*_1_ is before the vector at *t*_2_. Therefore, this paper encodes the position of the original data to solve the problem that the attention mechanism does not consider the time sequence.

Position coding is a method of quadratic representation of each time slot in the sequence with the position information of different time slots. As mentioned above, the self-attention mechanism is to extract the relationship between vectors at different times. It does not have the ability to learn time series information like recurrent neural network, so it needs to encode the position and actively input the time series information to the model. Location coding is a new representation vector that combines the information of time and the original time series. In this way, the model can learn the time position information of the original time series. The proposed position coding formula is as follows.

(5) $$\begin{array}{c} encoding=\left[\begin{array}{c} e_{1}\\ e_{2}\\ e_{3}\\ \vdots \\ e_{h} \end{array}\right]\text{,}\\ e_{i}=\left[\begin{array}{ccc} e_{i}^{1} & \cdots & e_{i}^{f} \end{array}\right]\text{,}\\ \begin{array}{c} e_{i}^{1}=e_{i}^{j},\left\{j=1,2,\cdots ,f\right\},\\ e_{i}^{1}=\frac{i-\mu }{\sigma },\\ \mu =\frac{1}{h}\overset{h}{\underset{i=1}{\sum }}i,\\ \sigma =\sqrt{\frac{1}{h}\overset{h}{\underset{i=1}{\sum }}{\left(i-\mu \right)}^{2}}. \end{array} \end{array}$$

Encoding represents the coding information of the whole input time window, *H* represents the length of the time series, and *f* represents the number of selected features; *e*_*i*_ represents the coding information vector at the *i*-th time in the input time window, *μ* represents the expectation of the length of the time series, and *σ* represents the standard deviation of the length of the time series.

### 2.4. Convolution Fusion

**Figure 9:**
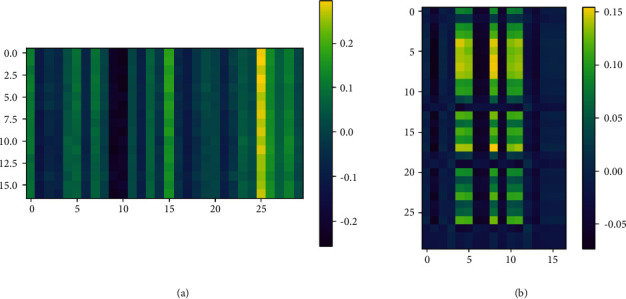
Spatio-temporal characteristic map; (a) spatial feature map; (b) time characteristic diagram.

After the original data is processed by the attention mechanism, the characteristic diagram of the data in time and space is obtained, as shown in Figure 9. Each line vector on the spatial feature graph represents the spatial state relationship between the original input line feature and all features; each line vector on the time feature graph represents the correlation between the features of each time moment and the features in the whole-time window. For the feature map, the convolution neural network is used to reduce the dimension and further extract the internal information of the data. Then RUL is predicted by two-layer fully connected neural network.

**Figure 10:**
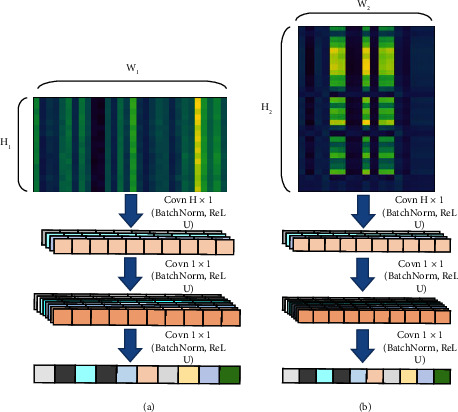
Convolution fusion structure. (a) CNN in spaital feature map. (b) CNN in time feature map.

In this paper, the feature map is convoluted in only one direction, so the feature vector can be fused into a specific value. Therefore, the high *h* of the feature map is reduced to 1, which plays a role in reducing the dimension. We call this convolution fusion, and Figure 10 shows the structure of the convolution fusion model.

## 3. Results and Discussion

### 3.1. Data Set Introduction

**Figure 11:**
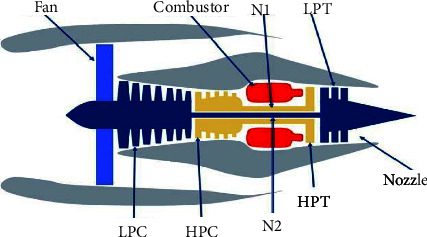
Engine structure diagram.

The data set used in the experiment is the C-MAPSS data set of NASA [19]. This data set is generated by the simulation software simulating the aeroengine. The schematic diagram of the simulated engine is shown in Figure 11, including high-pressure turbofan, high-pressure compressor, fan, nozzle, and burner. See Table 1 for a detailed description of the data set.

Table 1 shows that the data set is divided into four sub-data sets FD001, FD002, FD003, and FD004 according to different operating conditions and fault modes. Each sub-data is divided into training data set and test data set, which records three operation settings and 21 sensor data of scroll engine unit at each time. We use these indicators to divide spatio-temporal data. FD001 and FD002 sub-datasets contain one failure mode (HPC degradation), and FD003 and FD004 contain two degradation modes (HPC degradation and fan degradation); FD001 and FD003 have only one operating condition, and FD002 and FD004 have six operating conditions. Because the operation environment of FD002 and FD004 sub-dataset engine unit is complex and changeable, the prediction of RUL of FD002 and FD004 sub-dataset is more difficult.  Table 2 [20] lists the 21 sensors that monitor engine conditions.

### 3.2. Evaluating Indicator

In terms of remaining life prediction of the turbofan engine, many important journals [3, 7] references and the International Conference on fault prediction and health management [19] take score and root mean square error (RMSE) as the evaluation indexes of RUL prediction results. In order to enhance comparability, these two evaluation indexes are also used in this paper. The scoring function will have a larger penalty score for the predicted value with larger deviation; the root mean square error considers the real deviation between the predicted value and the actual value. The specific formula is as follows.

(6) $$\begin{array}{c} s=\overset{N}{\underset{i=1}{\sum }}s_{i},s_{i}=\left\{\begin{array}{c} e^{-\frac{d_{i}}{13}}-1,\text{for }d_{i}<0\\ e^{\frac{d_{i}}{10}}-1,\text{for }d_{i}\geq 0 \end{array}\right.\text{,}\\ d_{i}=predict_{i}-RUL_{i}\text{,}\\ \text{RMSE}=\sqrt{\frac{1}{N}\overset{N}{\underset{i=1}{\sum }}d_{i}^{2}}\text{.} \end{array}$$

predict_*i*_ represents the predicted value, RUL_*i*_ represents the real RUL value, and *N* represents the number of sample data.

**Figure 12:**
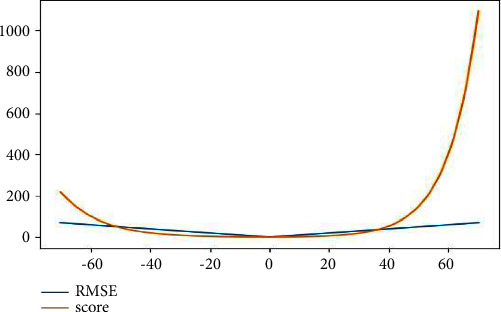
Evaluation index function.

In Figure 12 shows the relationship between RMSE and score; the abscissa value is the difference between the predicted value and the real value, and the ordinate is the specific value of RMSE and score. It can be seen that when the deviation between the predicted value and the real value is large, the value of score increases exponentially.

### 3.3. Experiment and Result Analysis

**Figure 13:**
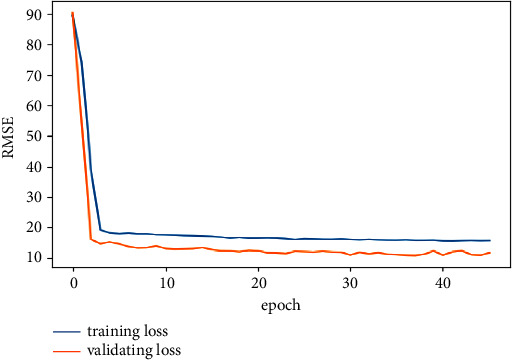
Training loss map.

In order to prevent over fitting of the model, we use dropout [21, 22] technology and verification set early stop strategy. The loss value in the training process is shown in Figure 13.

From Figure 13, it can be seen that the training of more than 40 epochs stopped after the model training. Because the use of early stop strategy avoids the data characteristics in the model over learning training set. At the same time, it can also be seen that during the training process, the RMSE of the verification set is less than that of the training set. Because the dropout technology is added to the full-connection layer. During training, some neural units of the full-connection layer will be inactivated, and when solving the RMSE of the verification set, all the inactivated neural units will be activated. In order to verify the impact of adopting the early stop strategy, we also trained 200 epochs for the model. The experimental results are shown in Table 3.

Table 3 shows that with the early stop strategy, the deviation between the RMSE of the FD001 training set and the FD001 test set is not large, and there is no fitting phenomenon. However, after 200 cycles of training the model directly, there is a large deviation between the RMSE of the FD001 training set and the FD001 test set, and there is a problem of overfitting. At the same time, the RMSE of the training model with early stop strategy is 10.89 on the FD001 training set, and the RMSE of the model with 200 training cycles is 8.44. The difference is in a reasonable range, and there is no obvious underfitting phenomenon. The prediction effect of the test set of the remaining three sub-data sets is shown in Table 4.

**Figure 14:**
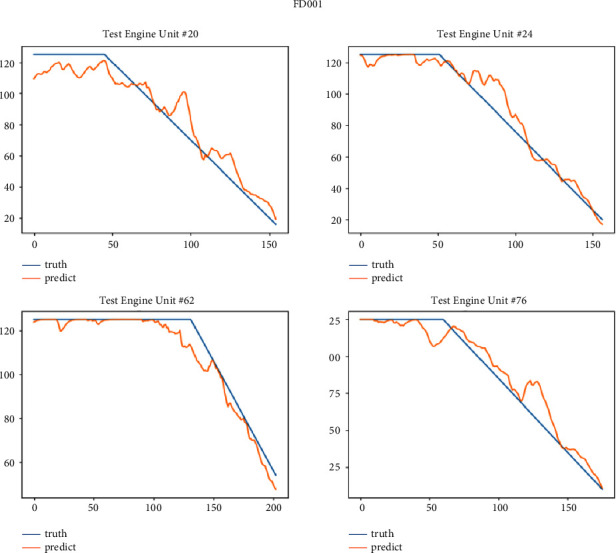
Sub-data set FD001 prediction RUL curve.

After the model is trained, we test it on the test set of four sub-data sets. This paper selects some engine units with two health stages for display. The FD001 sub-data set selects engine units 20, 24, 62, and 76; FD002 sub-data set selects 44, 62, 70 and 236 engine units; the FD003 sub-data set selects engine units 24, 78, 94 and 99; the FD004 sub-data set selects 213, 235, 244 and 248 engine units. Figures 11–14 show the prediction results of these engine units.

**Figure 15:**
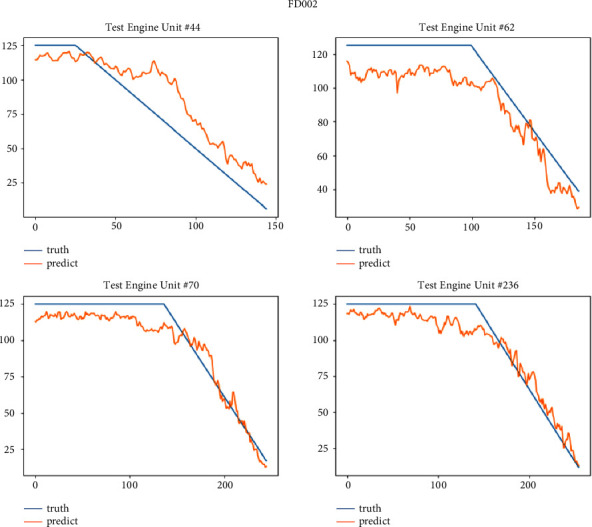
RUL curve of sub-dataset FD002 prediction.

**Figure 16:**
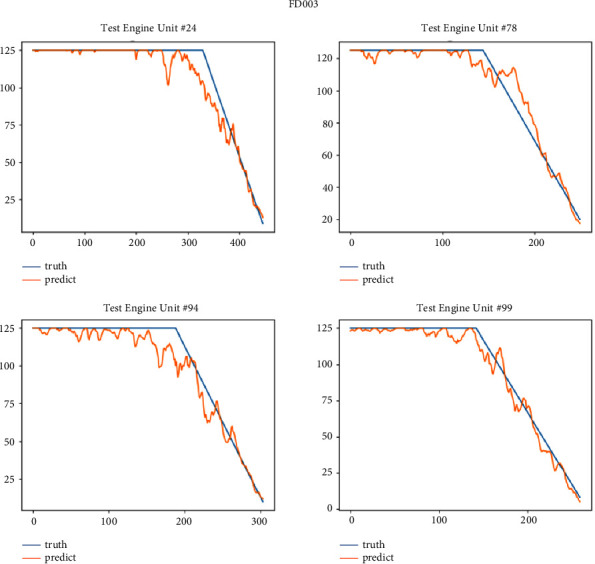
RUL curve of sub-dataset FD003 prediction.

**Figure 17:**
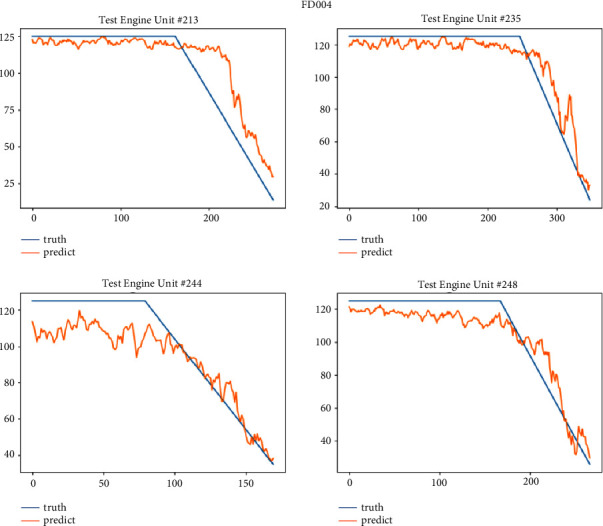
RUL prediction data set FD004 curve.

It can be seen from Figures 14to 17 that the predicted RUL curve presents a piecewise linear state, which truly reflects the degradation state of the real RUL. However, since FD001 and FD002 sub-datasets contain one failure mode (HPC degradation), FD003 and FD004 contain two degradation modes (HPC degradation and fan degradation); FD001 and FD003 have only one operating condition, and FD002 and FD004 have six operating conditions. The data distribution of FD001 and FD003 sub-datasets will be relatively regular. See Figures 3–6 for details. Therefore, the prediction accuracy of the model in the FD0001 and FD003 sub-datasets is higher than that in FD0002 and FD004 sub-datasets.

In order to verify the stable prediction accuracy of our proposed method, this paper tests the proposed model on the test set on each sub-data set, and compares the results with the prediction RUL method related to deep learning in recent years. See Table 4 for detailed comparison data.

As shown in Table 4, the spatio-temporal attention method proposed in this paper is superior to most methods in terms of RMSE and score. In the sub-data sets FD002 and FD004 under six different flight conditions, the spatio-temporal attention method reduces the RMSE to 18.10 and 17.00, and the score to 1339.59 and 2476.906, respectively. This reflects that the proposed spatio-temporal attention method still performs well and has strong robustness in the multi-traffic environment. Compared to the BiGRU-AS method, the spatio-temporal attention takes into account not only the relationship between time series but also the hidden states between sensors in spatial locations, which makes full-use of the data. At the same time, our method actively applies time series weights to the model, which can reduce the effect of data noise. Generally speaking, the spatio-temporal attention method has certain advantages over other methods in the table.

## 4. Conclusion

In this paper, the proposed attention mechanism model is used to learn the relationship between time series and the relationship between sensors in spatial position, and the RUL of a turbofan engine is predicted by convolution neural network fusion. All in all, this model addresses the problem of relying on human experts for feature selection and improves autonomy. At the same time, the attention mechanism can fill the gap between laboratory results and industrial practice, which can be an excellent parallel computing feature and improve the speed of model prediction in industrial practice. Finally, the proposed method can be fully embedded into the control and optimization integration framework to provide critical information about the system [8]. Through comparative analysis, the spatio-temporal attention mechanism method is superior to other methods in the evaluation indexes of RMSE and score. Especially in the multi-operation environment, the performance of this method is more stable than other methods. Of course, there are still some problems that need to be further explored. First, the potential harm caused by the situation that the predicted value is greater than the real value is relatively large, but at present, there is no good method to balance the relationship between reducing the predicted value and improving the prediction accuracy; second, there are many types of industrial data, but there may be some potential common features between degraded data. How to extract and use these common features to predict RUL and migrate to different scenarios is also a difficult problem; third, there are uncertainties and inaccuracies in available data, and how to eliminate these problems is also a difficulty in current research. At present, fuzzy logic [27] can generate the best fuzzy rule base in the learning process, which is a solution.

## Figures and Tables

**Table 1:** Data set introduction.

Sub-datasets	FD001	FD002	FD003	FD004
Engine units in the training dataset	100	260	100	249
Engine units in the test dataset	100	259	100	248
Fault modes	One (HPC degradation)	One (HPC degradation)	Two (HPC degradation, fan degradation)	Two (HPC degradation, fan degradation)
Conditions	One (sea level)	Six	One (sea level)	Six
Training samples	17731	48819	21820	57522
Test samples	100	259	100	248

**Table 2:** Sensor introduction.

Index	Description	Symbol
1	Total temperature at fan inlet	°R
2	Total temperature at LPC outlet	°R
3	Total temperature at HPC outlet	°R
4	Total temperature at LPT outlet	°R
5	Pressure at fan inlet	psia
6	Total pressure in bypass-duct	psia
7	Total pressure at HPC outlet	psia
8	Physical fan speed	rpm
9	Physical core speed	rpm
10	Engine pressure ratio (P50/P2)	—
11	Static pressure at HPC outlet	psia
12	Ratio of fuel flow to Ps30	Pps/psi
13	Corrected fan speed	rpm
14	Corrected core speed	rpm
15	Bypass ratio	—
16	Burner fuel-air ratio	—
17	Bleed enthalpy	—
18	Demanded fan speed	rpm
19	Demanded corrected fan speed	rpm
20	HPT coolant bleed	lbm/s
21	LPT coolant bleed	lbm/s

**Table 3:** Experimental results of different training strategies.

Method	FD001 training set		FD001 test set	
	RMSE	Score	RMSE	Score
Early stopping	10.89	371.14	**11.07**	**312.55**
200 epcohs	**8.44**	**202.26**	13.151	374.93

**Table 4:** Comparison of experimental results.

Method	FD001		FD002		FD003		FD004	
	RMSE	Score	RMSE	Score	RMSE	Score	RMSE	Score
Spatio-temporal attention	**11.07**	312.55	**18.10**	**1339.5**	**10.73**	329.95	**17.00**	**2476.90**
BiGRU-AS [6]	13.68	284	20.81	2454	15.53	428	27.31	4708
Ensemble ResCNN [9]	12.16	212.48	20.85	2087.77	12.01	**180.76**	24.97	3400.44
AdaBN-DCNN [10]	13.17	279	20.87	2020	14.97	817	24.57	3690
MS-DCNN [11]	11.44	**196.22**	19.35	3747	11.67	241.89	22.22	4844
Semi-supervised [23]	12.56	231	22.73	3366	12.1	251	22.66	2840
DCNN [24]	12.61	273.7	22.36	10412	12.64	284.1	23.31	12466
MODBNE [25]	15.04	334.23	25.05	5585.34	12.51	421.91	28.66	6557.62
DBN [26]	15.21	417.59	27.12	9031.64	14.71	442.43	29.88	7954.51
LSTM [25]	16.14	338	24.49	4450	16.18	852	28.17	5550
MLP [26]	16.78	560.59	28.78	14026.72	18.47	479.85	30.96	10444.35

## Data Availability

The data set used in the experiment is the C-MAPSS dataset of NASA. This dataset was generated with the C-MAPSS simulator. C-MAPSS stands for “Commercial Modular Aero-Propulsion System Simulation”,and it is a tool for the simulation of realistic large commercial turbofan engine data. For more information, please visit: https://github.com/uuuuf9/CMAPSSData.
